# CTHRC1: An Emerging Hallmark of Pathogenic Fibroblasts in Lung Fibrosis

**DOI:** 10.3390/cells13110946

**Published:** 2024-05-30

**Authors:** Zhussipbek Mukhatayev, Altynay Adilbayeva, Jeannette Kunz

**Affiliations:** Department of Biomedical Sciences, School of Medicine, Nazarbayev University, 5/1 Kerey and Zhanibek Khans St., 020000 Astana, Kazakhstan; zhussipbek.mukhatayev@nu.edu.kz (Z.M.); altynay.adilbayeva@nu.edu.kz (A.A.)

**Keywords:** biomarker, CTHRC1, extracellular matrix, collagen, TGF-β, idiopathic pulmonary fibrosis, post-COVID-19, pirfenidone, bexotegrast

## Abstract

Pulmonary fibrosis is a chronic, progressive, irreversible lung disease characterized by fibrotic scarring in the lung parenchyma. This condition involves the excessive accumulation of extracellular matrix (ECM) due to the aberrant activation of myofibroblasts in the alveolar environment. Transforming growth factor beta (TGF-β) signaling is a crucial driver of fibrogenesis because it promotes excessive ECM deposition, thereby leading to scar formation and lung damage. A primary target of TGF-β signaling in fibrosis is Collagen Triple Helix Repeat Containing 1 (CTHRC1), a secreted glycoprotein that plays a pivotal role in ECM deposition and wound repair. TGF-β transcriptionally regulates CTHRC1 in response to tissue injury and controls the wound healing response through functional activity. CTHRC1 may also play an essential role in re-establishing and maintaining tissue homeostasis after wound closure by modulating both the TGF-β and canonical Wnt signaling pathways. This dual function suggests that CTHRC1 regulates tissue remodeling and homeostasis. However, deregulated CTHRC1 expression in pathogenic fibroblasts has recently emerged as a hallmark of fibrosis in multiple organs and tissues. This review highlights recent studies suggesting that CTHRC1 can serve as a diagnostic and prognostic biomarker for fibrosis in idiopathic pulmonary fibrosis, systemic sclerosis, and post-COVID-19 lung fibrosis. Notably, CTHRC1 expression is responsive to antifibrotic drugs that target the TGF-β pathway, such as pirfenidone and bexotegrast, indicating its potential as a biomarker of treatment success. These findings suggest that CTHRC1 may present new opportunities for diagnosing and treating patients with lung fibrosis.

## 1. Introduction

**Figure 1:**
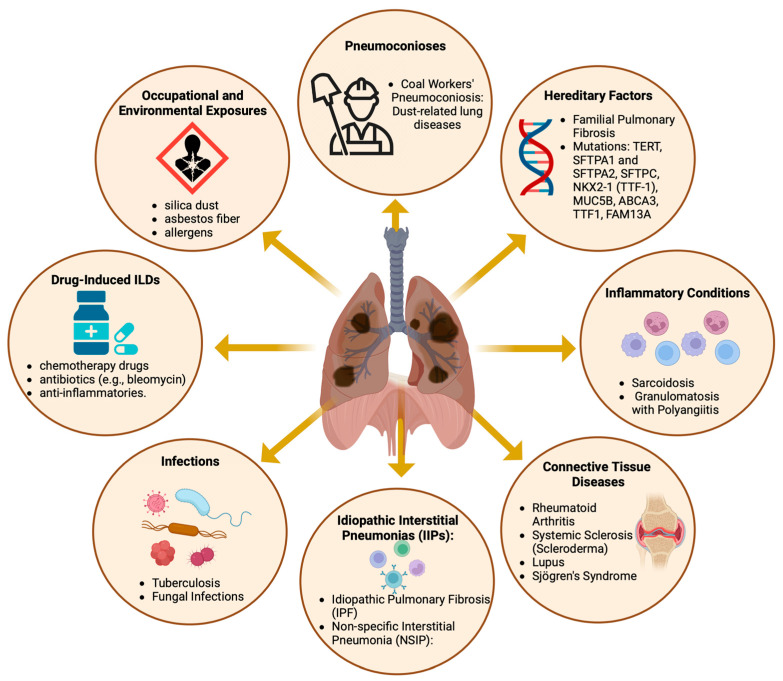
The causes and contributing factors of diffuse parenchymal lung diseases. Created with BioRender.com. URL accessed on 26 February 2024.

Diffuse parenchymal lung diseases encompass a diverse array of disorders (Figure 1), primarily characterized by progressive fibrosis of the pulmonary architecture, frequently culminating in respiratory failure [1,2]. Despite the varied etiologies of these pulmonary disorders, the majority exhibit aberrant fibrotic proliferation and infiltration of inflammatory cells as principal clinical manifestations, impacting the alveolar walls of the lungs. These pathological aberrations are predominantly observed in the lung interstitium, hence the alternate designation of interstitial lung diseases (ILDs) [3].

Idiopathic pulmonary fibrosis (IPF) is the most prevalent fibrotic ILD. It is characterized by the radiographic and histopathological pattern of usual interstitial pneumonia without an identifiable etiology or association with a known cause of pulmonary fibrosis [4]. IPF is chronic and irreversible and frequently results in respiratory failure and mortality. IPF has a greater incidence in males than in females and is more common among individuals aged 60 years and older [5]. In contrast, other ILDs typically present at a younger mean age (20 to 60 years) and display an equal distribution between the sexes [5,6]. In recent years, post-COVID-19 pulmonary fibrosis, a condition in which patients who have recovered from COVID-19 develop lung scarring and fibrotic changes leading to persistent respiratory symptoms and reduced lung function, has been described [7,8].

For classification and therapeutic considerations, ILDs are usually assigned to distinct disease categories (Figure 1), primarily based on the presence of an underlying medical condition (e.g., pulmonary fibrosis associated with rheumatoid arthritis (RA)), an inciting factor (e.g., pneumoconiosis and SARS-CoV-2 infection), or the absence of an identifiable etiology (e.g., IPF) [9,10,11,12]. Nevertheless, identifying and classifying patients with interstitial lung disease (ILD) is challenging, given the similarities in symptoms shared with other respiratory disorders, such as chronic obstructive pulmonary disease (COPD), asthma, and even heart failure. Advances in our understanding of the disease mechanisms that cause pulmonary fibrosis are expected to lead to the discovery of new biomarkers for the disease, which could enhance diagnostic options [13]. Moreover, these studies can potentially improve treatment strategies by identifying new molecular targets. Currently, the number of treatment options remains limited. To date, the FDA has approved only two medications for the treatment of IPF: pirfenidone, marketed as Pirespa, and nintedanib, available under the brand names Ofev and Vargatef [14,15]. Although these medications can slow the progression of the disease, they do not significantly increase patient survival and are frequently associated with severe adverse effects [14,16]. Thus, there is a medical need to identify and develop new diagnostic and therapeutic options.

Recent research has highlighted the role of CTHRC1, a secreted protein involved in ECM and tissue remodeling, as a biomarker for lung fibrosis and coexpressed proteins as a marker for disease diagnosis and the monitoring of pirfenidone treatment [17,18,19,20]. This review will focus on the pathophysiological characteristics of pulmonary fibrotic diseases and the potential role of CTHRC1 as a biomarker for identifying pathogenic fibroblasts involved in this disease.

## 2. Epidemiology of ILDs

Despite the relatively low incidence of specific fibrosing ILDs, these conditions collectively impact many patients. Current estimates indicate 76.0 ILD cases per 100,000 individuals in Europe and 74.3 cases per 100,000 individuals in the United States [21]. However, determining the exact number of people affected by different ILDs worldwide is difficult due to several factors, including difficulties in diagnosis, incorrect diagnosis, and the absence of uniform diagnostic practices in healthcare systems worldwide.

The most prevalent fibrotic ILD is IPF, with an estimated prevalence of 30.2 per 100,000 individuals [2,21]. IPF is inherently progressive, and after diagnosis, patients generally have a median lifespan of 2.5 to 3.5 years [22,23,24]. It is characterized by a steady decline in lung function, which eventually leads to permanent respiratory failure. Epidemiological research on North American and European demographics has shown that the number of new IPF cases ranges from 3 to 9 per 100,000 people per year, with a trend of increasing prevalence [25]. The significant risk factors for IPF are male sex and older age, particularly affecting those over 70 years of age [25,26,27].

Epidemiological research on ILDs distinct from IPF has been limited. Apart from IPF, the most frequently documented conditions include connective tissue disease-associated ILD (CTD-ILD), with an occurrence rate between 0.5 and 10.2 cases per 100,000 individuals; sarcoidosis, with 1.9 to 66.1 cases per 100,000 people; and ILD associated with systemic sclerosis (SSc-ILD), with a reported prevalence range of 7.2–33.9 and 13.5–44.3 per 100,000 individuals in Europe and North America, respectively [2,28]. Individuals with sarcoidosis and CTD-ILDs tend to be younger and more likely to be female and nonsmokers than those diagnosed with IPF [2,21], as illustrated in Figure 1. Similarly, individuals affected by SSC-ILD are predominantly female, with a female-to-male ratio of approximately 5:1. These studies revealed significant regional variations in reported estimates, with the highest prevalence in North America, followed by European populations [2,28]. However, it is unclear whether disparities may result from differences in diagnostic efficacy or truly reflect genetic, geographical, or demographic differences.

Recent investigations have revealed that lung fibrosis affects up to 70% of patients with rheumatoid arthritis (RA). This observation suggests that extra-articular manifestations of RA contribute to adverse patient outcomes in an estimated 5–10% of established RA patients [29,30,31].

## 3. Pathogenesis of Lung Fibrosis

**Figure 2:**
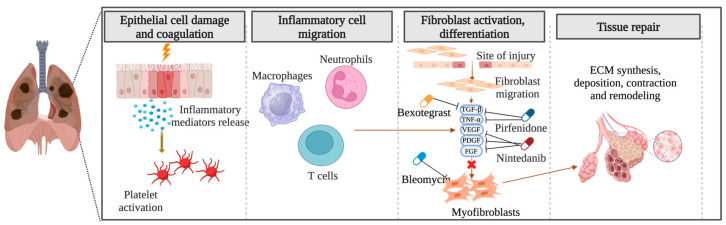
Pathogenesis of pulmonary fibrosis: Unraveling the cascade of inflammatory responses and fibrotic remodeling. Created with BioRender.com. URL accessed on 29 February 2024.

The precise pathogenesis of fibrosing ILDs remains incompletely understood. In the context of pulmonary fibrosis, diverse disease-specific triggers initiate abnormal cascades of inflammatory responses, culminating in the synthesis, deposition, contraction, and remodeling of ECM components and the formation of fibrotic tissue (Figure 2). Numerous aspects of the etiology of specific diseases and the criteria demarcating normal wound healing from fibrotic progression remain obscure. Notwithstanding the myriad etiologies underlying pulmonary fibrosis, advanced stages exhibit shared pathophysiological mechanisms [32,33].

The prevailing theory suggests that repetitive damage to the alveolar epithelium caused by exposure to a range of harmful environmental factors, including cigarette smoke, gastric acid, air pollution, and viruses, leads to aberrant wound healing with excessive deposition of ECM by mesenchymal cell populations in the lung parenchyma [34,35,36,37,38]. This, in turn, may result in successive scarring and the development of interstitial fibrosis in individuals with a predisposition for this disease due to their genetic makeup [39]. As fibrosis progresses, the ability of the lungs to exchange gases diminishes, leading to dyspnea, the onset of respiratory failure, and, ultimately, death [40].

Genetic studies have identified common and rare susceptibility loci associated with pulmonary fibrosis [41]. Many genes that confer an increased risk of disease development are associated with alveolar epithelial cells or inflammatory responses. For instance, a common polymorphism in the *MUC5B* promoter, which is implicated in airway clearance and microbial host defense, has been correlated with an increased risk of IPF, RA with ILD [42], and chronic hypersensitivity pneumonitis [43], but not with SSc-ILD or sarcoidosis. Patients with IPF, RA with ILD, and chronic hypersensitivity pneumonitis exhibit telomere shortening and alterations in telomere-related genes, including TERT and *TERC* [42,43]. Certain rare genetic variations, including mutations affecting telomeres, are also strongly associated with progressive disease [42]. In addition, HLA status has been implicated as a predisposing factor [41].

In addition to shared genetic risk factors, the early stages of various ILDs exhibit heterogeneous and overlapping molecular pathways [9]. In IPF, an unidentified disruption of alveolar epithelial cell integrity might trigger pathology through interactions between endothelial cells and myofibroblasts [6]. In sarcoidosis, only a small fraction of patients develop fibrosis in response to granulomatous inflammation caused by a putative, persistent, and yet-to-be-identified etiology [44].

The causes of either Ssc, a chronic autoimmune connective tissue disease, or SSc-ILD are currently unknown. A substantial fraction (50%) of people living with SSc also have ILD, which is a significant contributor to morbidity and mortality [45]. SSc-ILD involves a complex disease process in which different factors and pathways, including immunological abnormalities, endothelial dysfunction resulting in small vessel vasculopathy, and fibroblast dysfunction resulting in excessive collagen production leading to fibrosis, are affected [32,46,47]. Investigations of specific patients with Ssc-ILD have indicated that the disease is driven by repetitive epithelial injury, leading to varied inflammatory responses that may foster a profibrotic environment and cytokine milieu characterized by the expression of TGF-β, connective tissue growth factor (CTGF), platelet-derived growth factor (PDGF), and interleukins (ILs), such as IL-6 [48,49].

Although the precise mechanisms underlying the various forms of ILDs may differ, the pathological activation of fibroblasts is a common driver of fibrogenesis [50]. Fibroblasts in nonpathological adult tissues typically remain quiescent. However, they undergo activation in response to injury and play a central role in fibrogenesis through dysregulated production of ECM, differentiation into myofibroblasts, secretion of profibrotic mediators, and interaction with immune cells [50]. Fibroblasts typically become activated in response to damaged alveolar type II epithelial (AT2) cells, which are mainly responsible for epithelial repair upon injury [51]. AT2 cells contribute to wound repair by secreting large amounts of cytokines and growth factors (CTGF, PDGF, and interleukins) that promote pulmonary fibroblast recruitment, proliferation, and differentiation into myofibroblasts [51]. These cells also secrete inflammatory mediators that lead to the recruitment and activation of immune cells [51]. AT2 cells and immune cells further release TGF-β, which is a key factor in fibrosis development. Specifically, TGF-β promotes the differentiation of fibroblasts into myofibroblasts [52]. Myofibroblasts are characterized by enhanced contractility and the ability to produce increased amounts of ECM components, including various collagens and fibronectin [52].

In the case of nonrepetitive tissue injury, the wound repair response is transient until normal tissue homeostasis is restored, at which time fibrogenic myofibroblasts are removed through apoptosis, senescence, or dedifferentiation and reprogramming. However, in recurrent or severe injury cases, such as those observed in chronic inflammatory diseases, ECM components persistently accumulate. The excessive accumulation of collagen and other ECM components induces alterations in the fibrillar collagen network, resulting in increased tissue stiffness within the lung parenchyma. This process disrupts the normal architecture of lung tissue, impairs gas exchange, and results in progressive and irreversible scarring of the lungs and, ultimately, the loss of lung function [2,40].

In addition to the secretion of collagens, myofibroblasts contribute to tissue remodeling through the expression and secretion of matrix metalloproteinases (MMPs) and tissue inhibitors of metalloproteinases (TIMPs), which regulate the turnover and degradation of the ECM. In fibrosis, the balance between MMPs and TIMPs is often disrupted, which further sustains pathogenic ECM accumulation [53]. Fibroblasts and myofibroblasts also secrete various cytokines and growth factors, including TGF-β, PDGF, and CTGF, which perpetuate their activation and recruit other fibroblasts and immune cells. This leads to a positive feed-forward cycle that promotes the formation of a profibrotic environment [54].

**Figure 3:**
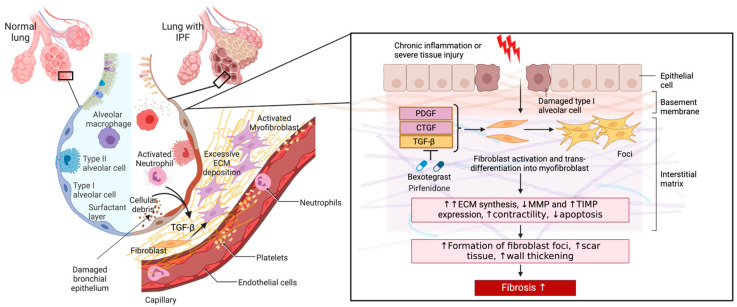
The role of pathogenic fibroblasts in IPF. A comparison of a healthy lung and one with IPF is shown. Alveolar damage through repetitive insults and dysregulated inflammatory cells leads to inflammation, alveolar remodeling, and the release of active TGF-β and other profibrotic mediators (arrows) into the interstitium that enhance fibroblast-myofibroblast transition and ECM secretion. Excessive ECM deposition results in a scarred interstitium, alveolar wall thickening, and the development of fibrosis. Figure created with BioRender.com. URL accessed on 29 February 2024.

Pathogenic fibroblasts in pulmonary fibrosis also proliferate abnormally and exhibit resistance to apoptosis. This increases the number of cells contributing to ECM production and leads to the formation of fibroblastic foci, clusters of activated fibroblasts, and myofibroblasts associated with the fibrotic destruction of lung architecture observed in IPF (Figure 3) [55,56,57].

Given their central role in the pathogenesis of lung fibrosis, targeting myofibroblasts and their interactions with other cells therefore offers potential therapeutic avenues for treating lung fibrosis. However, the source of pathogenic fibroblasts in pulmonary fibrosis has remained a matter of debate. Prevailing theories regarding the origin of these cells suggest that they originate from (1) resident fibroblasts, (2) fibrocytes, which are bone marrow-derived fibroblast progenitors, and (3) alveolar epithelial cells undergoing epithelial–mesenchymal transition (EMT) [58,59]. Importantly, as outlined further below, single-cell RNA-sequencing studies recently identified a pathogenic subpopulation of fibroblasts that appear to differentiate from alveolar fibroblasts and were present only in fibrotic lesions in murine and human lungs, including those from IPF, ILD, and SSc patients [20,60]. Notably, these cells are characterized by their high expression of collagen and *CTHRC1*, a secreted protein involved in ECM remodeling during wound repair [61]. Single-cell transcriptomic and lineage-tracing studies further suggested that the CTHRC1-positive fibroblast subset produces the pathological ECM that drives fibrogenesis. This review highlights the current evidence linking CTHRC1-positive fibroblasts to the pathogenesis of pulmonary fibrosis.

## 4. CTHRC1—Structural Characteristics, Expression, and Disease Relationship

CTHRC1 was initially identified as a secreted ECM glycoprotein pivotal for orchestrating vascular remodeling and tissue repair processes while promoting cell migration [62,63,64]. CTHRC1 is a C1q/tumor necrosis factor-α-related protein family member that is highly conserved among vertebrates. CTHRC1 encompasses an N-terminal signal peptide, a short collagen triple helix composed of 12 Gly-X-Y repeats that may mediate multimerization, and a COOH-terminal globular C1q domain [62]. Secreted CTHRC1 exists primarily as a dimer (56 kDa) and a trimer (84 kDa), but can likely also form multimers of the trimeric CTHRC1, resulting in higher molecular weight complexes (168 kDa and 252 kDa) [65]. Moreover, secreted CTHRC1 might gain biological activity through further proteolytic processing at the N-terminus [66].

CTHRC1 is expressed mainly during development but is essentially undetectable in healthy adults. During skeletal development, abundant CTHRC1 expression is observed in cartilage primordia, growth plate cartilage, bone matrix, and periosteum. CTHRC1 is restricted to the bone matrix and periosteum in the adult body. However, its expression is transiently upregulated in various mesenchymal-derived cells during growth and injury-induced tissue repair [63,65]. Pathological expression of CTHRC1 has also been detected in the matrix of calcifying atherosclerotic plaques and mineralized bone tissues [67].

Furthermore, elevated CTHRC1 expression has been implicated in the pathophysiology of numerous cancerous, autoinflammatory, and autoimmune disorders. Accordingly, CTHRC1 was identified as a novel oncogene aberrantly overexpressed in various malignant tumors, exhibiting associations with bone metastasis and unfavorable patient outcomes [68,69,70,71]. CTHRC1 gene expression is also upregulated in RA and has recently been reported to be a potential biomarker for disease diagnosis [72]. Cthrc1 RNA and protein levels were significantly increased in a mouse arthritis model. In this mouse model, immunohistochemistry analysis further revealed highly elevated Cthrc1 expression in fibroblast-like synoviocytes at the invasive edge of the pannus [73]. CTHRC1 protein levels were also significantly greater in the serum of RA patients than in that of healthy individuals [72]. Similar to what was found in mice, CTHRC1 expression was elevated in two subpopulations of synoviocytes isolated from the pannus of RA patients, one of which was strongly linked to disease pathology [74]. Therefore, CTHRC1 may contribute to cartilage degradation in individuals with RA by modulating the migration and invasion of a pathological synoviocyte subpopulation. While CTHRC1 overexpression in fibroblasts confers pathologic effects in RA, there is also evidence for anti-inflammatory effects in a collagen antibody-induced arthritis murine model, suggesting that CTHRC1 can also play a protective role [75]. Thus, CTHRC1 may play multifaceted roles in various pathological conditions depending on its expression and mechanism of action in specific cell types, tissues, and disease conditions.

Apart from its documented involvement in cardiovascular disease, cancer, and RA, an expanding body of research also associates CTHRC1 with fibrotic conditions such as systemic lupus erythematosus, muscular dystrophies, and fibrosis in the lung, heart, liver, and skin [20,72,76,77,78,79,80,81]. In systemic lupus erythematosus, significant increases in CTHRC1 levels in the plasma and serum of patients have been observed [72,77,81]. In systemic lupus erythematosus, aberrant CTHRC1 expression has been linked to the dysregulation of immune responses and tissue remodeling, contributing to the development of fibrotic manifestations [77]. Similarly, in muscular dystrophies, CTHRC1-mediated alterations in fibroblast activity and extracellular matrix turnover exacerbate tissue fibrosis, leading to progressive muscle degeneration [78]. Furthermore, in pulmonary fibrosis, emerging evidence suggests a pivotal role for CTHRC1 in promoting fibroblast activation, myofibroblast differentiation, and collagen deposition within the lung parenchyma [61,76]. Understanding the signaling pathways regulated by CTHRC1 in these fibrotic conditions holds promise for identifying novel therapeutic targets to mitigate fibrosis progression and improve clinical outcomes. Therefore, investigating the intricate involvement of CTHRC1 in fibrotic pathogenesis may enhance our understanding of disease mechanisms and highlight potential avenues for targeted therapeutic interventions.

## 5. Molecular Pathways and Mechanism of Action of CTHRC1

The primary physiological function of CTHRC1 is associated with its involvement in tissue remodeling following injury. This includes regulating collagen matrix deposition, wound healing, and cell migration as part of the TGF-β signaling pathway [76,82]. Accordingly, in many tissues, CTHRC1 expression overlaps considerably with the expression of interstitial collagens and members of the TGF-β family. Furthermore, CTHRC1 transcription is induced by TGF-β and BMP-4 [83].

The physiological role of CTHRC1 in the TGF-β pathway was first characterized in wounded cardiac arteries, where TGF-β plays a pivotal role in orchestrating tissue repair processes following injury [82,84,85]. This is achieved through the upregulation of collagen and fibronectin synthesis, the expression of genes involved in ECM remodeling, and cell proliferation and migration mediated by the activation of SMAD2/3 complexes. This results in increased ECM synthesis and deposition and enhanced proliferation and migration of smooth muscle cells [82,84,85]. The TGF-β signaling pathway activates the expression and secretion of different collagens, such as COL1A, fibronectin, and *CTHRC1* [82].

**Figure 4:**
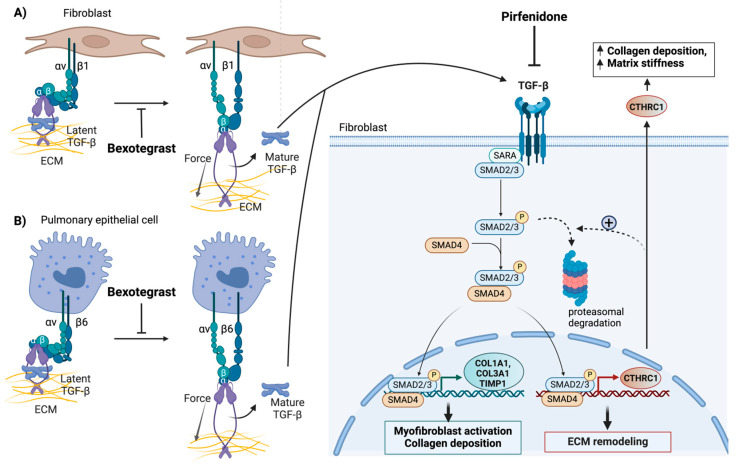
Systemic inhibition of TGF-β signaling in the fibrotic lung by pirfenidone and localized inhibition of TGF-β signaling in the fibrotic niche by bexotegrast (**A**,**B**). Both pirfenidone and bexotegrast affect the synthesis of CTHRC1 and other profibrotic genes, such as *COL1A1*, *COL3A1*, and *TIMP1*. This reduces collagen deposition, tissue stiffness, and the invasive properties of collagen- and CTHRC1-positive profibrotic fibroblasts. Bexotegrast acts as a dual integrin αVβ6 and αVβ1 inhibitor that attenuates pulmonary fibrosis by inhibiting the integrin-dependent activation of latent TGF-β. Pirfenidone globally blocks the synthesis and activation of TGF-β. Only the canonical TGF-β signaling pathway is depicted. The proposed negative feedback effect of CTHRC1 on TGF-β signaling, which involves the degradation of phospho-SMAD2/3 by the proteasome (indicated by broken arrows), is highlighted. This feedback mechanism may terminate the normal wound-healing response. The pathological effects of elevated CTHRC1 on fibrosis, induced by repetitive injury, may involve a different mechanism of action. The figure was created with BioRender.com. URL accessed on 29 February 2024.

The exact role of CTHRC1 in the TGF-β-mediated wound healing response is currently unknown. However, comparative studies between Cthrc1-deficient and wild-type mice suggest that CTHRC1 can transiently accelerate wound healing, leading to enhanced migration and increased M2 macrophage infiltration [86,87] (Figure 4). This is, at least, partially achieved by inducing localized collagen degradation [86,87].

While CTHRC1 is necessary for TGF-β-induced wound repair in response to injury, increased CTHRC1 expression subsequently attenuates TGF-β signaling to terminate the wound healing process [82,83]. As a result, sustained activation of CTHRC1 expression exerts an antagonistic effect on TGF-β signaling, leading to reduced phosphorylation of SMAD2/3 in vascular cells [83]. The inhibition of TGF-β-sensitive reporters by CTHRC1 and the observed reduction in phospho-Smad2/3 protein levels due to proteasomal degradation in response to sustained overexpression of CTHRC1 collectively support this notion (Figure 4). Therefore, CTHRC1 may play a dual role in TGF-β signaling, promoting the re-establishment and maintenance of proper tissue homeostasis.

While CTHRC1 appears to modulate TGF-β signaling both positively and negatively during nonrepetitive tissue injury, its role during chronic injury seems to switch to that of a TGF-β effector. For example, exogenously added CTHRC1 was shown to upregulate the proliferation of Cthrc^−/−^ murine fibroblasts and their differentiation into myofibroblasts, which promoted wound contraction and excessive ECM deposition [87].

Existing evidence also indicates that both the canonical and noncanonical Wnt pathways converge at the CTHRC1 level [76]. CTHRC1 is reportedly stabilized through N-glycosylation by dolichol-phosphate N-acetylglucosamine-phosphotransferase 1 (DPAGT1), which enhances its promigratory function [64]. Activation of Wnt/β-catenin signaling induces the expression of DPAGT1 [88], establishing a connection between Wnt/β-catenin signaling and CTHRC1 [64]. CTHRC1, in turn, activates noncanonical Wnt5A signaling, thereby acting as a switch between the canonical and noncanonical Wnt pathways. Notably, the actions of CTHRC1 in the noncanonical Wnt and TGF-β signaling pathways may be closely related. CTHRC1 derived from cardiac fibroblasts, activated through the canonical TGF-β1-Smad2/3 pathway, may enhance wound healing and reduce the risk of cardiac rupture following myocardial infarction by activating the noncanonical Wnt signaling pathway [89].

In addition to its role in the TGF-β and noncanonical Wnt pathways, CTHRC1 promotes accelerated wound repair in mice by regulating the Notch pathway [86]. CTHRC1 may also contribute to IL-1β-induced apoptosis of chondrocytes by activating the JNK1/2 pathway [90].

## 6. CTHRC1 as a Potential Biomarker in Pulmonary Fibrosis

It is widely acknowledged that the ECM composition within healthy lungs significantly differs from that under pathological conditions, such as those associated with chronic lung disease. Although it is difficult to pinpoint the precise modifications in the ECM experimentally, mouse models of lung fibrosis and technological advances such as lineage-tracing experiments, cell labeling methods, and better reporter systems, along with the advent of single-cell RNA sequencing and spatial transcriptomics approaches, have recently allowed us to better define the alterations in lung composition that occur in fibrotic lung diseases.

The first experimental evidence that CTHRC1 plays a central role in fibrotic pathology was obtained in murine models of lung fibrosis. In vitro cultured fibroblasts were further used, in which *CTHRC1* was either upregulated or downregulated, or cells were treated with exogenous CTHRC1 to determine the role of CTHRC1 in TGF-β signaling and ECM remodeling. However, these approaches have provided conflicting evidence regarding the precise role (pathogenic or protective) of CTHRC1 under different fibrotic conditions.

Accordingly, in a global Cthrc1 knockout murine model, bleomycin administration resulted in increased immune responses and decreased lung function, most likely due to the rapid accumulation of collagen in the lung in the absence of *Cthrc1* expression. Therefore, this study suggested that Cthrc1 inhibits TGF-β signaling to restore normal tissue homeostasis after bleomycin-induced lung injury [80]. The same study confirmed the negative effect of Cthrc1 on TGF-β and, indirectly, on canonical Wnt signaling using isolated and cultured Cthrc1^−/−^ murine fibroblasts [80]. The findings of this study aligned with observations from a murine model of vascular fibrosis, wherein transient expression of *Cthrc1* in adventitial fibroblasts following injury was correlated with reduced collagen deposition in carotid arteries [82,83]. Moreover, injection of recombinant Cthrc1 was reported to inhibit TGF-β-stimulated collagen deposition and mitigate fibrotic changes in a bleomycin-induced murine model of dermal fibrosis [91].

In contrast, recent advancements in single-cell genomics and lineage-tracing studies support the notion that CTHRC1 expressed in specific fibroblast subsets plays a pathological rather than a protective role in fibrogenesis. Analyses using single-cell transcriptomics revealed heterogeneity among fibroblasts present in the fibrotic lungs of both mice and IPF patients and emphasized the pivotal involvement of CTHRC1-positive myofibroblasts in pulmonary fibrosis. In a study by Tsukui et al., a subset of fibroblasts characterized by elevated levels of collagens, particularly COL1A1 and COL3A1, along with increased *CTHRC1* expression, was identified in both mice and IPF patients [20]. Notably, these COL1A1+ and CTHRC1+ cells were termed ‘pathological fibroblasts’ due to their heightened migratory capacity to infiltrate the lung and their localization at the periphery of fibrotic lesions. These findings were validated in an independent study, which additionally demonstrated that inhibition of the CTHRC1/HIF-1α axis hampers the initiation and progression of interstitial lung fibrosis [92].

In a subsequent study, Tsukui et al. utilized lineage tracing in transgenic mice to monitor *Cthrc1*-expressing lung fibroblasts following bleomycin-induced pulmonary fibrosis [93]. They validated the findings of their single-cell transcriptomics analysis, confirming that *Cthrc1*+ cells exhibited heightened expression of ECM genes, including *Col1a1*. Moreover, the elimination of *Cthrc1*-expressing fibroblasts in lung fibrosis using *Cthrc1*-CreER mice attenuated hydroxyproline, a marker of collagen deposition, following bleomycin administration [93]. Taken together, these results suggest that these *Cthrc1*-expressing cells drive ECM deposition and may promote fibrosis.

Thus, CTHRC1 may serve as a marker of a specific subset of activated fibroblasts implicated in the development of fibrosis. This fibroblast subset may be transitional and develop from alveolar fibroblasts [93,94], which provides a niche for maintaining alveolar type 2 cells in uninjured lungs. These cells undergo sequential stages of activation and differentiation after lung injury and appear to be the main drivers of fibrogenesis. Accordingly, lineage-tracing studies in mice showed that inflammatory cytokines initially induce chemokine-producing inflammatory fibroblasts from alveolar fibroblasts, which can differentiate into activated CTHRC1-positive fibrotic fibroblasts in response to TGF-β [93].

Recent single-cell RNA sequencing studies also identified CTHRC1 as a marker of activated fibroblasts driving the development of SSc. Previous studies implicated the TGF-β-responsive genes periostin (POSTN) and cartilage oligomeric matrix protein (COMP) as biomarkers of activated TGF-β signaling. These studies showed that increased *POSTN* and *COMP* expression in the skin is correlated with the modified Rodnan skin score, which has predictive value for disease progression [95,96]. In dermal tissues from SSc patients and skin tissues from mice with bleomycin-induced fibrosis, *CTHRC1* expression, along with the expression of *POSTN* and other pathological ECM components, was elevated in a fibroblast subset marked by leucine-rich repeat-containing G-protein-coupled receptor 5 (LGR5) and associated with SSc pathology [97].

Comparable CTHRC1-positive pathologic fibroblast subpopulations associated with human cardiac [98,99] and hepatic fibrosis [99], frozen shoulder capsule fibrosis [100], keloids [26], and dermal fibrotic lesions that result from abnormal wound healing in response to skin trauma were identified using single-cell transcriptomics analysis.

Thus, single-cell genomics and lineage-tracing data support the notion that CTHRC1-expressing fibroblast subpopulations are pathologic. These studies highlight the potential of CTHRC1 as a diagnostic biomarker for fibrotic diseases and a potential therapeutic target.

## 7. CTHRC1: A Potential Prognostic Marker for Severe Lung Complications in COVID-19 Patients

The coronavirus disease 2019 (COVID-19) pandemic caused by the SARS-CoV-2 virus has caused a range of symptoms in affected individuals, from asymptomatic cases to severe conditions that result in lung fibrosis and potentially fatal respiratory failure [7]. In COVID-19-affected lung tissue, there is a considerable increase in the number of cells from the monocyte-macrophage lineage [101,102], and these cells are predominantly located in the extravascular lung tissue, which is primarily ‘interstitial’ rather than ‘alveolar’ [102,103]. These highly active macrophage populations express inflammatory markers and genes linked to tissue repair and fibrogenesis [61,104].

Patients experiencing long-term COVID-19 infection display enduring disruptions in immune responses even eight months postinfection. This is evident through persistent increases in activated CD14+CD16+ monocytes and plasmacytoid dendritic cells compared to those in controls. Additionally, sustained elevation in type I (IFNβ) and type III (IFNλ1) interferons persists, which, along with other factors such as pentraxin 3, IFNγ, IFNλ2/3, and IL-6, forms a combination indicative of long-haul COVID-19, with accuracies reaching 78.5% to 81.6% [105]. These elements, often tied to acute and severe disease, imply a delayed or ineffective resolution of inflammation in these patients. Conversely, overly intense inflammatory responses may precipitate irreversible lung fibrosis, causing significant respiratory function impairment. The expression of markers, such as lipocalin-2, matrix metalloprotease-7, and hepatocyte growth factor, closely aligns with inflammation severity and impaired pulmonary function [106]. The capacity of SARS-CoV-2 to alter immune homeostasis mechanisms that impact tissue inflammation likely underlies persistent lung injury. The decline in alveolar macrophages, which are integral for lung integrity, in severe COVID-19 may stem from damage to AT2 cells, which also express ACE2 receptors that the virus targets [107,108,109]. In COVID-19 fatalities, lung analyses have revealed inflammation-associated AT2 cell states that hinder proper regeneration, coupled with pathogenic fibroblasts expressing CTHRC1, possibly driving rapid pulmonary fibrosis progression [110]. TGF-β and epithelium-derived IL-6 could be implicated in this fibrotic process [20,61,111]. Macrophages are involved in the immunopathology observed in fatal COVID-19 patients, and profibrotic macrophages, particularly those containing interleukin 1 beta (IL-1β), can hinder epithelial repair [61,112]. Notably, a subpopulation of CTHRC1-positive pathological fibroblasts was increased in lungs affected by COVID-19 [20,101] and was associated with the progression of pulmonary fibrosis in COVID-19 patients [61,110,113]. Researchers identified four fibroblast clusters—adventitial, alveolar, intermediate pathological, and pathological. Remarkably, the latter two cell clusters exhibited substantial expansion in COVID-19 lungs compared to controls and were characterized by the expression of *CTHRC1*, *COL1A1*, and *COL3A1* [61,110,113]. These fibroblasts have previously been identified as pathologic drivers of fibrosis in patients with IPF [20]. These cells produce the highest levels of type 1 collagen and have an enhanced capacity to migrate and colonize the lung [20]. The increased risk of developing fibrosis could be attributed to the emergence of these CTHRC1-positive fibroblast populations and their close relationship with macrophages [101,102].

A recent study revealed the overexpression of profibrotic genes, including collagen and *POSTN*, in COVID-19 patients. This study further confirmed a significant increase in the expression of *CTHRC1*, a marker for myofibroblasts, which colocalize with regions of high alpha-smooth muscle expression [114,115,116]. Importantly, pulmonary fibrosis can develop and persist even after a patient fully recovers from COVID-19, emphasizing the need for diagnostic biomarkers and long-term treatment options to slow the progression of the disease [117]. The number of CTHRC1-positive pathological fibroblasts was also increased in a study of short postmortem interval specimens from patients with lethal COVID-19 [61].

These findings suggest that CTHRC1 may serve as a diagnostic biomarker for identifying patients who experience severe COVID-19 symptoms and are at risk of developing post-COVID-19 pulmonary fibrosis.

## 8. Potential Clinical Implications

As outlined above, several studies have identified CTHRC1 as a biomarker delineating a specific subpopulation of pathogenic fibroblasts. The elevated expression of *CTHRC1* in these profibrotic fibroblasts positions CTHRC1 as a potential pivotal player in the progression of pulmonary fibrosis. Consequently, the following question arises: does targeting these fibroblasts exert any discernible impact on disease pathogenesis or progression? Indeed, supporting this hypothesis, CTHRC1-positive fibroblasts are responsive to treatment with two antifibrotic agents: pirfenidone and bexotegrast.

Pirfenidone is an antifibrotic agent approved by the Food and Drug Administration (FDA) for managing IPF [118]. The mechanism of action of pirfenidone in lung fibrosis involves its ability to modulate multiple pathways implicated in fibrogenesis. Primarily, pirfenidone inhibits the synthesis of profibrotic factors such as TGF-β and tumor necrosis factor-alpha (TNF-α), thereby attenuating the excessive accumulation of ECM components such as collagen [50,119]. Notably, pirfenidone suppresses the fibrotic activity of patient-derived fibrotic fibroblasts by negatively regulating CTHRC1 through its effect on TGF-β [18]. Accordingly, pirfenidone suppressed TGF-β-induced *CTHRC1* expression and attenuated CTHRC1-dependent collagen gel contraction and chemotaxis.

Conversely, TGF-β stimulation increased *CTHRC1* expression and secretion specifically in fibrotic fibroblast lines derived from patients but not in control fibroblasts, indicating that CTHRC1-positive fibrotic fibroblasts may elicit an elevated response to pirfenidone treatment. These findings are consistent with the results obtained in bleomycin-induced lung fibrosis rodent models, in which pirfenidone was shown to affect *CTHRC1* expression [17]. Thus, CTHRC1 could be a dominant target for the antifibrotic effects of pirfenidone on fibrotic lung fibroblasts. Consequently, the response to pirfenidone treatment can potentially be predicted by evaluating CTHRC1 levels in myofibroblasts, providing an avenue for identifying novel treatment options for lung fibrosis [18].

Since CTHRC1-positive fibroblasts are implicated in the progression of fibrosis in COVID-19 patients, these findings raised the possibility of including pirfenidone within a standard treatment protocol to improve the outcome of post-COVID-19 lung fibrosis patients. Accordingly, recent studies have suggested that treating COVID-19 patients with pulmonary fibrosis with pirfenidone, particularly in the early stages of fibrosis, can improve patient outcomes, thus offering a promising therapeutic approach [120,121,122].

Bexotegrast (PLN-74809) is a newly developed selective inhibitor of αvβ1 and αvβ6 integrins [123]. Significantly, recent data from Pliant Therapeutics, which developed bexotegrast, showed that this drug targets a subgroup of CTHRC1-positive myofibroblasts responsible for excessive collagen production in pulmonary fibrosis [124]. Studies utilizing single-nuclei RNA sequencing of human lung tissue affected by fibrosis and treated with bexotegrast showed decreased expression of *CTHRC1* and other genes associated with fibrogenesis and the TGF-β pathway in these particular myofibroblasts [123,124]. Notably, bexotegrast treatment also decreased the population of CTHRC1-positive myofibroblasts [124]. These studies highlight CTHRC1-positive myofibroblasts as critical contributors to fibrogenesis and suggest that they are targets of therapeutic drugs, such as pirfenidone or bexotegrast, that can mitigate lung fibrosis.

A common denominator of the antifibrotic effects of pirfenidone and bexotegrast is their inhibition of TGF-β activity. The principal therapeutic action of pirfenidone is based on suppressing myofibroblast differentiation stimulated by TGF-β. Pirfenidone systemically inhibits TGF-β synthesis and activation and has been shown to reduce lung function decline and mortality in IPF patients. However, while clinical studies have shown that it reduces the decrease in forced vital capacity [15], pirfenidone is ineffective at reversing fibrosis or preventing disease progression [15,18]. Furthermore, pirfenidone systemically inhibits TGF-β, thus posing a risk of toxicity and adverse reactions due to the multifaceted roles of TGF-β in maintaining tissue homeostasis.

Bexotegrast is a newly developed drug for the treatment of patients with IPF that has recently received fast-track designation from the FDA and orphan drug designation from the European Medicines Agency (EMA). Bexotegrast is a selective dual inhibitor of αvβ1 and αvβ6 integrins. These integrins can additively promote the activation of latent TGF-β during wound healing and tissue remodeling [123,125,126] (Figure 4). While expressed at low levels in normal tissue, αvβ6 and αvβ1 integrins are upregulated in fibroblasts and alveolar epithelial cells, respectively, in IPF (Figure 4). Indeed, integrin αvβ1 and αvβ6 have both been identified as direct transcriptional targets of TGF-β [127,128]. Clinical studies have shown that bexotegrast can effectively attenuate latent TGF-β activation and type I collagen gene expression (Figure 4) in fibrotic human and mouse lung tissue slices at doses significantly lower than those of pirfenidone [123,124]. Thus, bexotegrast is expected to enhance the efficacy of therapeutic interventions while limiting toxicity by specifically targeting localized pathologic TGF-β signaling.

Taken together, systemic (pirfenidone) and localized (bexotegrast) inhibition of TGF-β reduced pro-fibrotic gene expression within a unique cell population of fibroblasts highly expressing *COL1A1* and *CTHRC1*. This finding suggests that *CTHRC1* can potentially be used to monitor the effects of antifibrotic drugs or combination therapies on this population of disease-relevant fibroblasts. Although the specific source and regulatory mechanism of these profibrotic fibroblasts still require additional studies, the successful attenuation of TGF-β-mediated *CTHRC1* expression or ablation of CTHRC1-positive fibroblasts by pirfenidone or bexotegrast further provides proof-of-concept that the targeting of CTHRC1-positive fibroblasts could offer novel therapeutic strategies to mitigate TGF-β-driven fibrosis and improve patient outcomes.

## 9. Future Directions

Accumulating evidence indicates that CTHRC1 plays a significant role in the pathogenesis of diffuse parenchymal lung diseases, with implications for diagnosis and treatment. The current understanding of CTHRC1 is consistent with its modulatory effects on fibroblast proliferation and collagen matrix remodeling mediated by the TGF-β signaling pathway. Nevertheless, there are discrepancies between mouse/in vitro and single-cell transcriptomics/lineage-tracing studies that necessitate further investigation to determine the precise role (protective or pathologic) of CTHRC1 in diverse fibrotic diseases.

Such studies will require improved genetic models, including inducible *Cthrc1* knockout mice, as well as drugs, such as bexotegrast, to deplete Cthrc1-positive fibroblast subtypes in various fibrotic diseases. Moreover, currently unidentified tissue-specific cell–cell interactions and microenvironmental factors may govern the expansion and differentiation of CTHRC1-positive fibroblasts in different fibrotic diseases [93]. Conventional knockout or bleomycin murine models and in vitro studies may not accurately recapitulate the complex and dynamic cellular network in the fibrotic niche that governs the molecular dysregulation of CTHRC1. Single-cell genomics and lineage-tracing studies that can identify heterogeneous cell populations are likely needed to provide better insight into the dynamic and complex fibrotic microenvironment.

The precedence for this stems from the examination of TGF-β and *CTHRC1* expression in keloids. Investigations utilizing Cthrc1^−/−^ mouse models and treatment with exogenous Cthrc1 indicated a protective role of Cthrc1 in keloids. Increased levels of Cthrc1 were associated with the attenuation of TGF-β signaling and the inhibition of collagen deposition [129]. Similarly, enhanced *Cthrc1* expression [130] or treatment of keloid fibroblasts with recombinant Cthrc1 [129] decreased collagen I synthesis in both normal skin fibroblasts and keloid fibroblasts in vitro by inhibiting TGF-β/Smad pathway activation. In contrast, single-cell RNA sequencing studies of human keloid tissue samples revealed the crucial involvement of local macrophages in the expansion and differentiation of CTHRC1-positive fibroblasts [26]. This effect was mediated by macrophage-secreted SPP1 (secreted phosphoprotein 1, also known as osteopontin) and enhanced pathogenic ECM deposition in fibrotic lesions [26]. These results highlight the crosstalk between stromal and myeloid cells as a significant area for future studies.

Moreover, a recent study employing longitudinal single-cell and lineage-tracing experiments identified secreted frizzled-related protein 1 (SFRP1) as a key modulator of TGF-β1-driven fibroblast to myofibroblast differentiation in pulmonary fibrosis [94]. Using a bleomycin mouse model, the authors demonstrated that TGF-β1 downregulated *Sfrp1* expression in noninvasive transitional fibroblast cells and induced their switch to highly invasive Cthrc1+/Spp1+ myofibroblasts. *Sfrp1*, an antagonist of the Wnt pathway [131,132], was expressed in the early stages after bleomycin injury and acted as an inhibitor of TGF-β1-driven myofibroblast phenotypes in fibrogenesis.

Taken together, these studies indicate that further single-cell genomics approaches, including epigenomic studies and systematic lineage-tracing analyses, are warranted to track *CTHRC1*-expressing cells. Such approaches are expected to clarify the specific origins and roles of these cells within different tissue microenvironments and to identify candidate regulators of differentiation. Moreover, comparative analyses of normal and fibrotic tissues using these methods are likely to provide additional insights into the mechanisms by which CTHRC1 influences normal wound healing as opposed to fibrotic diseases.

## 10. Conclusions

The information discussed in this review highlights the potential of CTHRC1 as a biomarker for the early detection of pulmonary fibrosis and as a possible target for developing novel therapeutic strategies. Recent studies have underscored the importance of CTHRC1 in pulmonary fibrosis and its regulatory role in maintaining the integrity of the lung matrix. The potential disruption of CTHRC1-related processes might contribute to the abnormal proliferation of fibroblasts and their transformation into pathogenic phenotypes. In addition, other research has indicated the role of CTHRC1 in ECM remodeling in fibrotic diseases in different tissues, including hepatic and cardiac fibrosis.

*CTHRC1* alone or as part of a profibrotic gene expression signature could serve as a marker that could aid in developing more effective diagnostic tools, preventive measures, or targeted therapeutic interventions for the progression of fibrosis and inflammation in patients. CTHRC1 could offer new avenues for the early stratification of patients, allowing more effective targeted treatment options. 

## Abbreviations

AT2	alveolar type II epithelial cell
C1q	complement component 1q
CD14/16	cluster of differentiation 14/16
COL1A	collagen type I alpha 1 chain
COL3A1	collagen type III alpha 1 chain
COMP	cartilage oligomeric matrix protein
COPD	chronic obstructive pulmonary disease
CTD	connective tissue disease
CTHRC1	collagen triple helix repeat containing 1
CTGF	connective tissue growth factor
DPAGT1	dolichyl-phosphate N-acetylglucosaminephosphotransferase 1
ECM	extracellular matrix
EMT	epithelial-mesenchymal transition
FDA	Food and Drug Administration
HIF-1α	hypoxia-inducible factor 1 alpha
IFN	interferon
IL	interleukin
ILD	interstitial lung disease
IPF	idiopathic pulmonary fibrosis
JNK	Jun N-terminal kinase
LGR5	leucine-rich repeat-containing G-protein-coupled receptor 5
MMP	matrix metalloprotease
MUC5B	mucin 5B, oligomeric mucus/gel-forming
NIH	National Institute of Health
PDGF	platelet-derived growth factor
POSTN	periostin
RA	rheumatoid arthritis
SFRP1	secreted frizzled-related protein 1
SMAD	suppressor of mothers against decapentaplegic
SPP1	secreted phosphoprotein 1
SSc	systemic sclerosis
TERC	telomerase RNA component
TERT	telomerase reverse transcriptase
TGF	transforming growth factor
TIMP	tissue inhibitor of metalloproteinase
TNF-α	tumor necrosis factor-alpha
Wnt	wingless
